# Supplementary figures and images for: Prosurvival long noncoding RNA PINCR regulates a subset of p53 targets in human colorectal cancer cells by binding to Matrin 3
Source: eLife. 2017 Jun 5;6:e23244. doi: 10.7554/eLife.23244 (PMC5470874; doi:10.7554/eLife.23244)

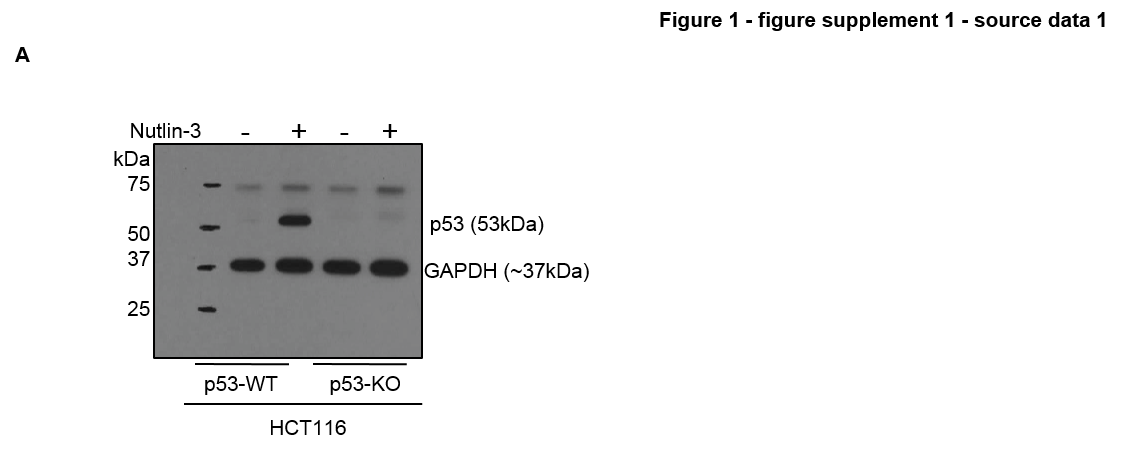

Supplement: Figure 1—figure supplement 1—source data 1. — DOI: http://dx.doi.org/10.7554/eLife.23244.005 [file elife-23244-fig1-figsupp1-data1.docx]

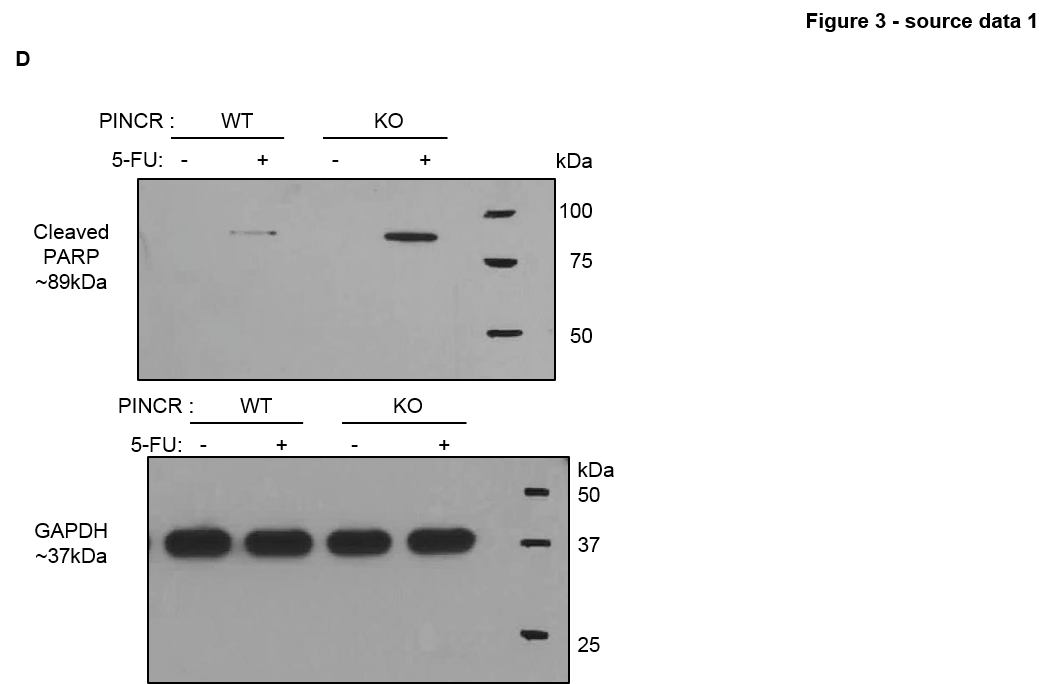

Supplement: Figure 3—source data 1. — DOI: http://dx.doi.org/10.7554/eLife.23244.020 [file elife-23244-fig3-data1.docx]

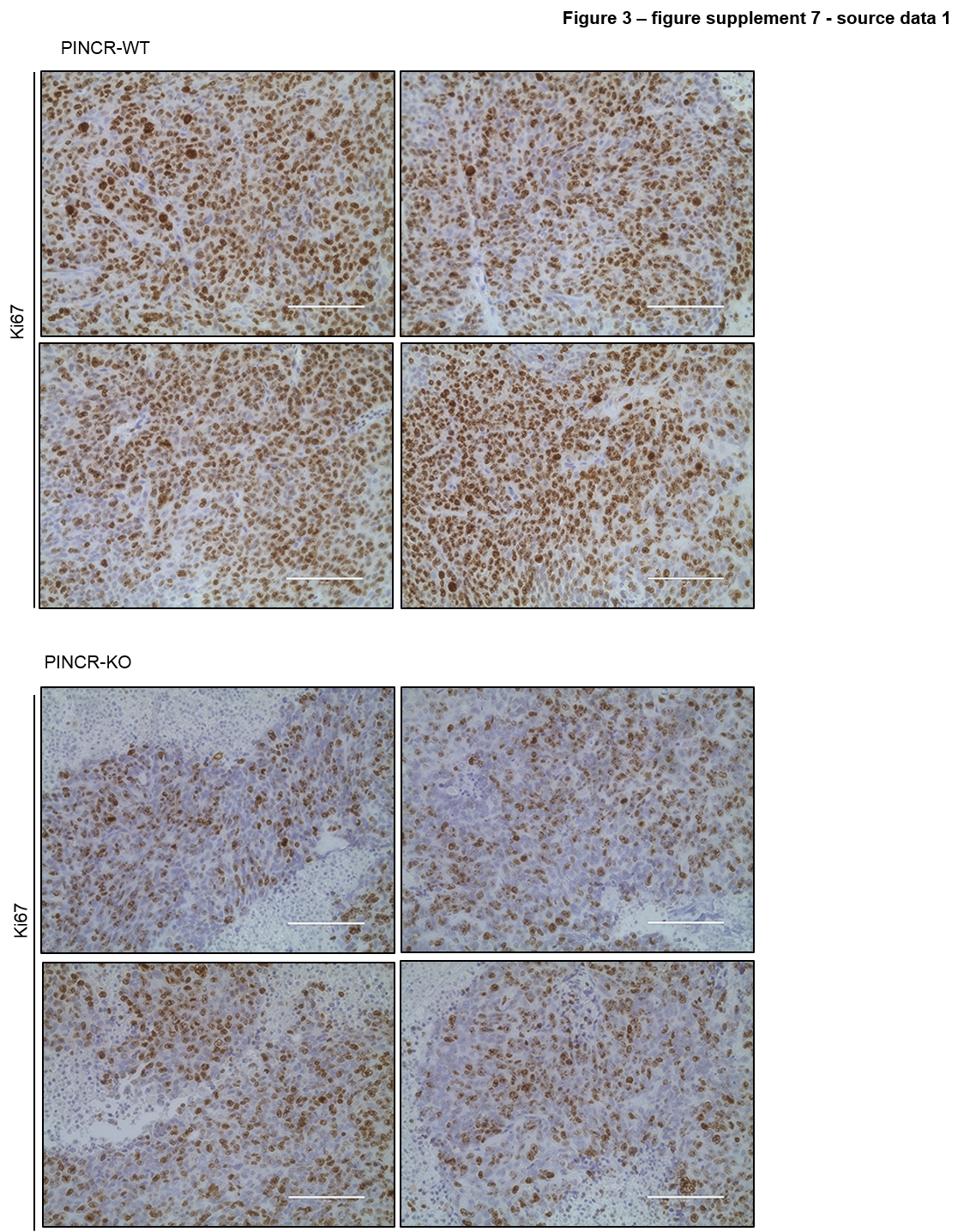

Supplement: Figure 3—figure supplement 7—source data 1. — DOI: http://dx.doi.org/10.7554/eLife.23244.028 [file elife-23244-fig3-figsupp7-data1.docx]

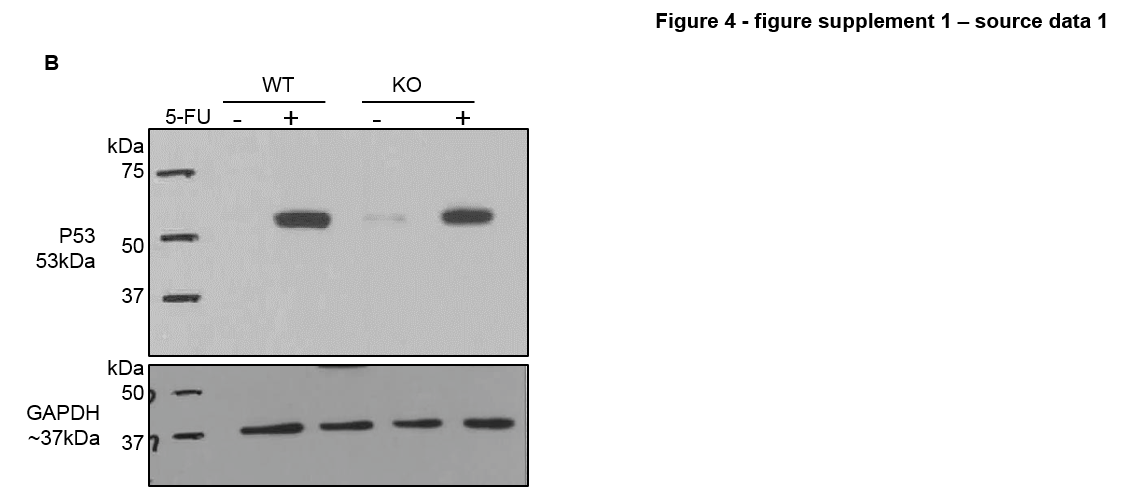

Supplement: Figure 4—figure supplement 1—source data 1. — DOI: http://dx.doi.org/10.7554/eLife.23244.031 [file elife-23244-fig4-figsupp1-data1.docx]

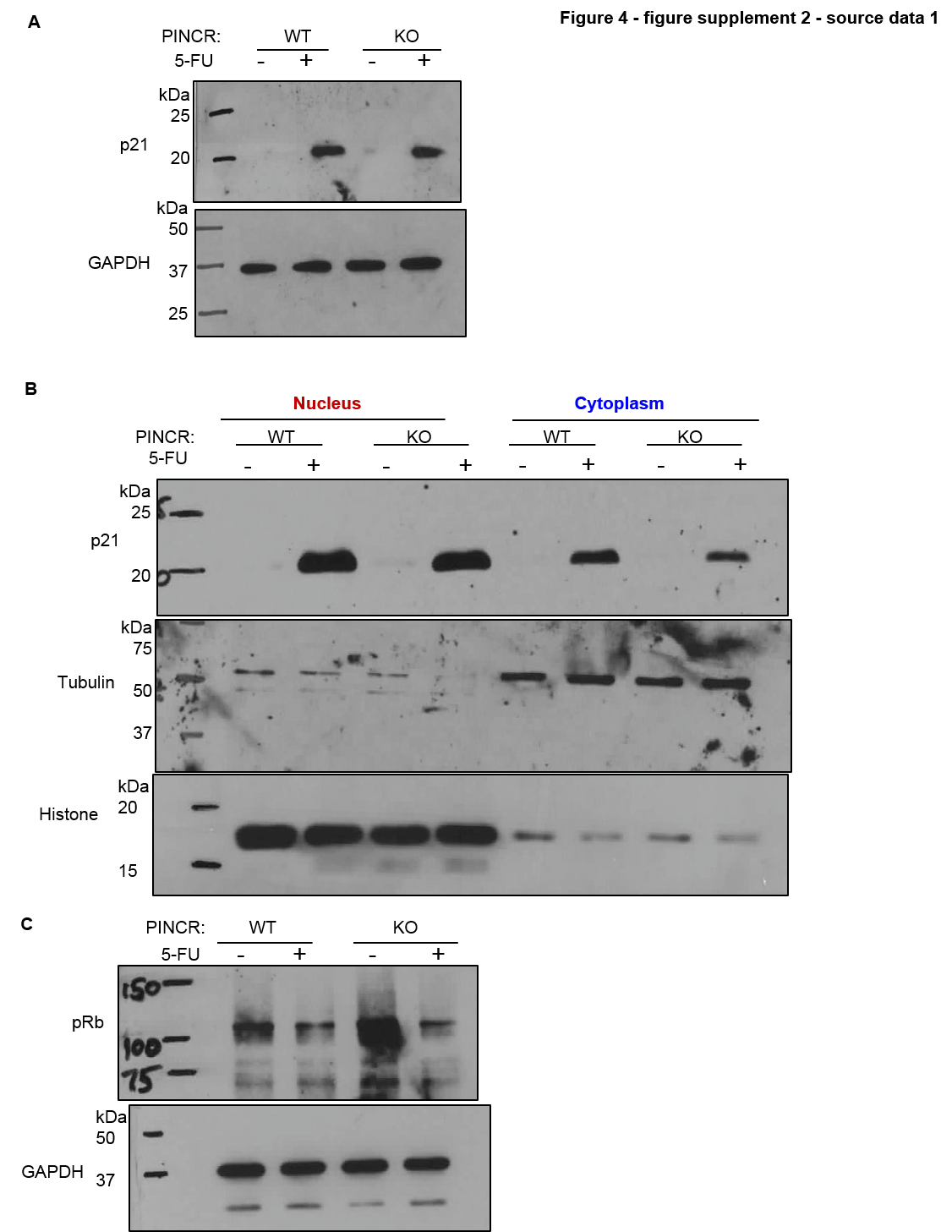

Supplement: Figure 4—figure supplement 2—source data 1. — DOI: http://dx.doi.org/10.7554/eLife.23244.033 [file elife-23244-fig4-figsupp2-data1.docx]

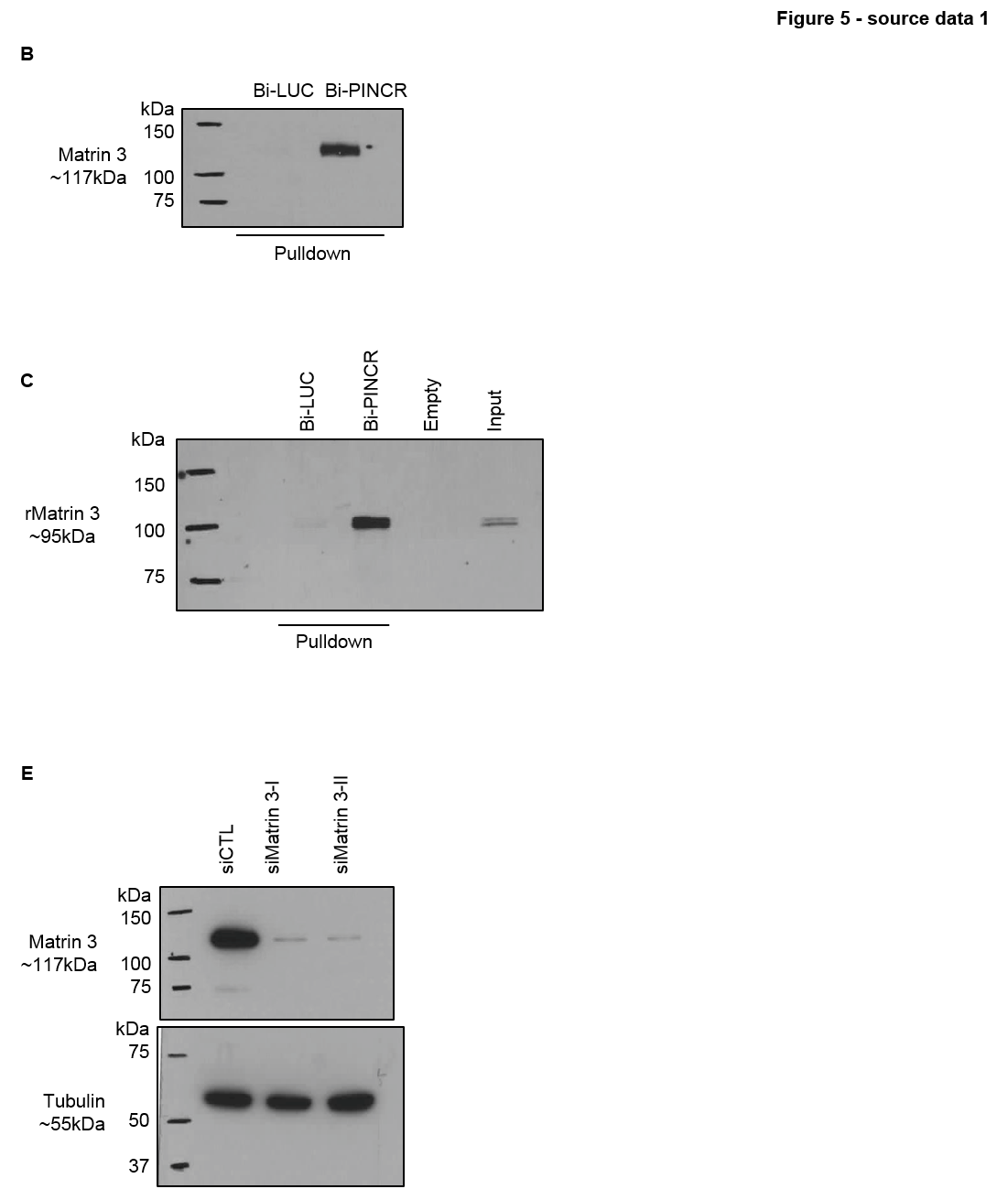

Supplement: Figure 5—source data 1. — DOI: http://dx.doi.org/10.7554/eLife.23244.039 [file elife-23244-fig5-data1.docx]

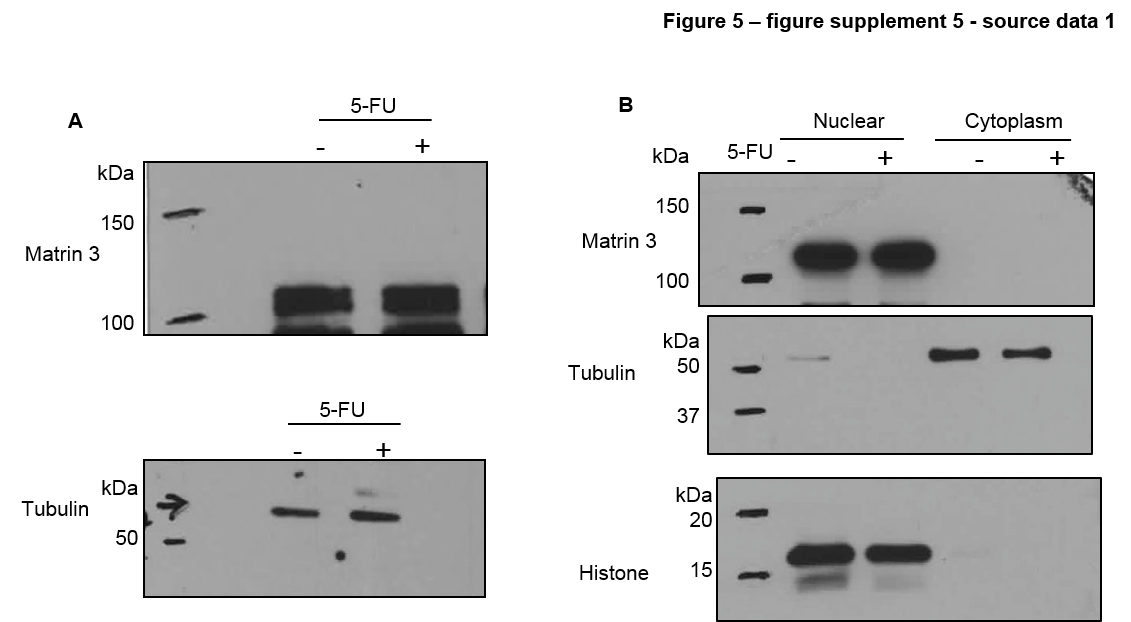

Supplement: Figure 5—figure supplement 5—source data 1. — DOI: http://dx.doi.org/10.7554/eLife.23244.045 [file elife-23244-fig5-figsupp5-data1.docx]

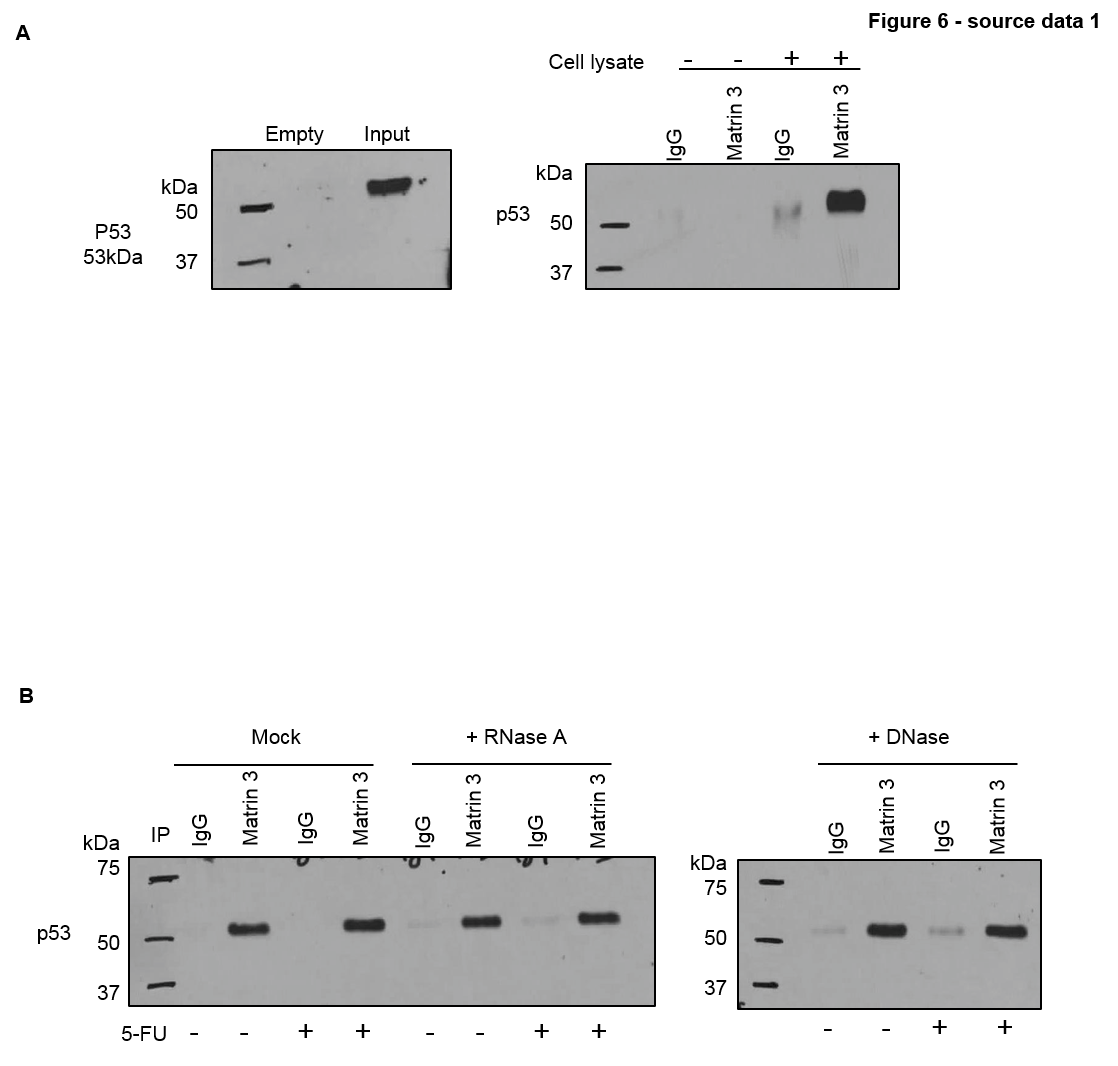

Supplement: Figure 6—source data 1. — DOI: http://dx.doi.org/10.7554/eLife.23244.047 [file elife-23244-fig6-data1.docx]

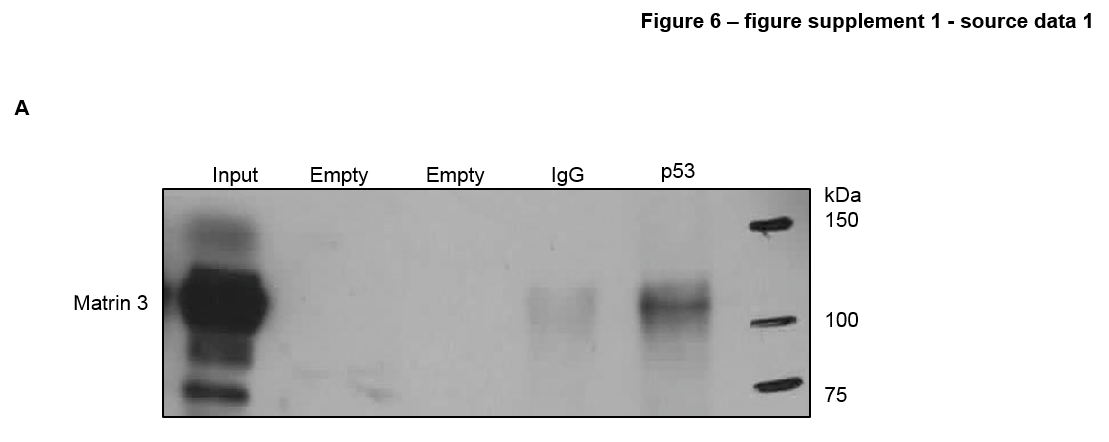

Supplement: Figure 6—figure supplement 1—source data 1. — DOI: http://dx.doi.org/10.7554/eLife.23244.049 [file elife-23244-fig6-figsupp1-data1.docx]

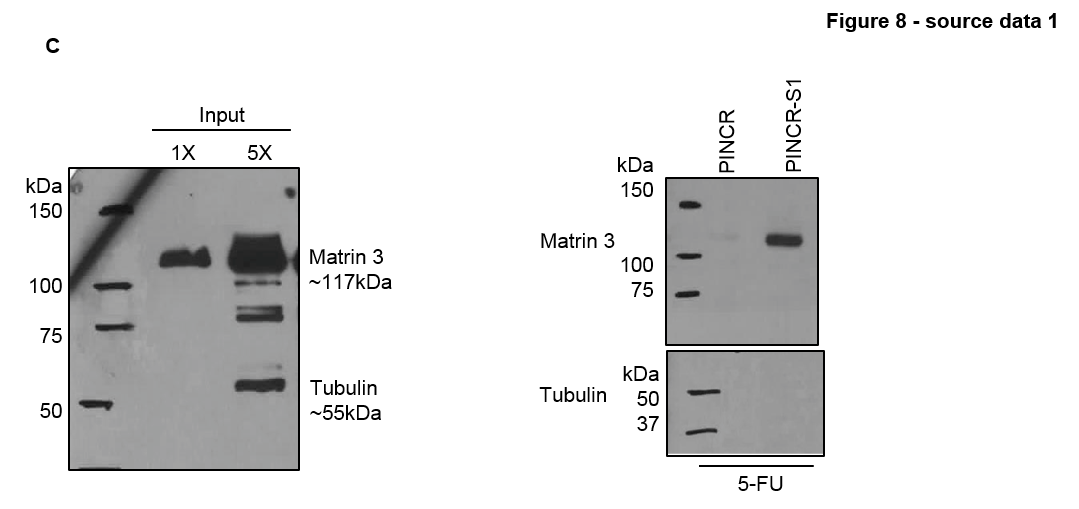

Supplement: Figure 8—source data 1. — DOI: http://dx.doi.org/10.7554/eLife.23244.055 [file elife-23244-fig8-data1.docx]

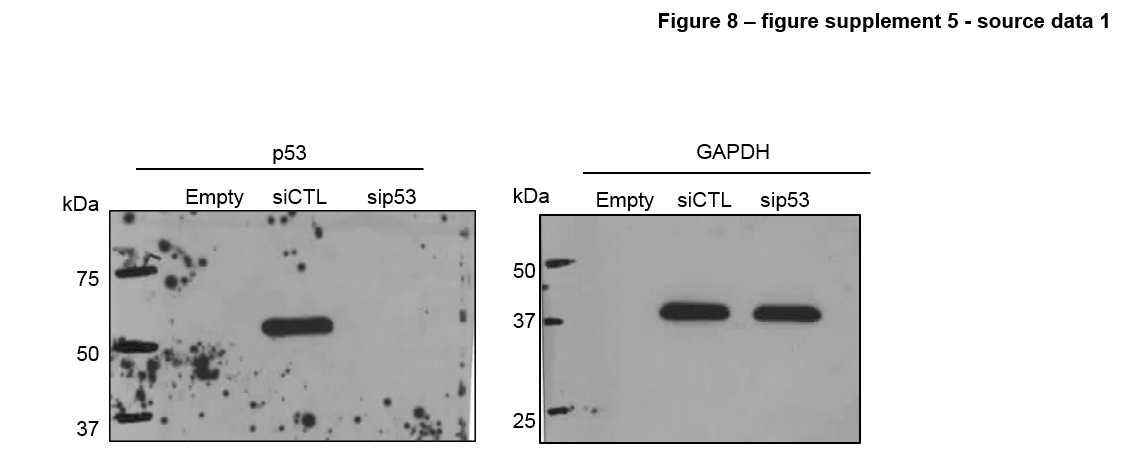

Supplement: Figure 8—figure supplement 5—source data 1. — DOI: http://dx.doi.org/10.7554/eLife.23244.061 [file elife-23244-fig8-figsupp5-data1.docx]
