# Supplementary material for: Prosurvival long noncoding RNA PINCR regulates a subset of p53 targets in human colorectal cancer cells by binding to Matrin 3
Source: eLife. 2017 Jun 5;6:e23244. doi: 10.7554/eLife.23244 (PMC5470874; doi:10.7554/eLife.23244)
Supplement: Figure 1—figure supplement 6—source data 1. — DOI: http://dx.doi.org/10.7554/eLife.23244.011 [file elife-23244-fig1-figsupp6-data1.docx]

**Multiple sequence alignment of mature *PINCR* transcript**

**Figure 1 - figure supplement 6 - source data 1**

Human ATTCAATCTGTGAGGTGGATGCGTGTTGGAAGGACCCCTTTTGGTTTCTTTTTGCTCAGA

Chimp ATTCAATCTGTGAGGTGGATGCGTGTTGGAAGGACCCCTTTTGGTTTCTTTTTGCTCAGA

Gorilla ATTCAATCTGTGAGGTGGACGCATGTTGGAAGGACCCCTTTTGGTTTCTTTTTGCTCAGA

Orangutan ATTCAATCTGTGAGGTGGA-GCGTGTTGGAAGGACCCGTTTTGGTTTCTTTTTGCTCAGA

Gibbon ATTCAATCTGTGAGGTGGATGCGTGTTGGAAGGACTCCTTTTGGTTTCTTTTTGCTCAGA

Crab-eating ATTCAATCTGTGAGGTGGAAGCCTGTTGGAAGGACCTCTTTTGGTTTCTTTTTGCTCAGA

Rhesus ATTCAATCTGTGAGGTGGAAGCCTGTTGGAAGGACCTCTTTTGGTTTCTTTTTGCTCAGA

Marmoset ATTCAATCTGTAGGGTGGGAGCCTGATGGAAGGACCGCTTTTGGGTT-TTCTTGCTCAGA

*********** ***** ** ** ********* ****** ** ** *********

Human GC---TTTCTTTTAATAAATTCCGCTCTCCTCACCTTTCAATGTGTCCGTATGCCTAATT

Chimp GC---TTTCTTTTAATAAATTCCGCTCTCCTCACCTTTCAATGTGTCCGTATGCCTAATT

Gorilla GC---TTTCTTTTAATAAATTCCGCTCTCCTCACCTTTCAATGTGTCTGTATGCCTAATT

Orangutan GC---TTTCTTTTAATAAATTCCGCTCTCCTCACCTTTCAATGTGTCCGTATGCCTAATT

Gibbon GC---TTTCTTTTAATAAATTCCACTCTCCTCACCTTTCAATGTGTCTGTATACCTAACT

Crab-eating GC---TTTCTTTTAATAAATTCCGCTCTCCTCACTTTTCAATGTGTCCATGTGCCTAATT

Rhesus GC---TTTCTTTTAATAAATTCCGCTCTCCTCACTTTTCAATGTGTCCATGTGCCTAATT

Marmoset GCTTTTTTTTTTTAATAAACTCAGCTCTCCTCACCTTTCAATGTGTCTGTGTGCCTAATT

** *** ********** ** ********** ************ * * ***** *

Human TTTCCTGGTCCTGTGACAAGAACCT-GATTTTAGCTGAACTAAGGAGCGAAAGATCCTGG

Chimp TTTCCTGGTCCTGTGACAAGAACCTGGATTTTAGCTGAACTAAGGAGCGAAAGATCCTGG

Gorilla TTTCCTGGTCTTGTGACAAGAACCTGGATTTTAGCTGAACTAAGGAGCTAAAGATCCTGG

Orangutan TTTCCTGGTCTTGTGACAAGAACCTGGATTTTAGCTGAACTAAAGAGCAAAAGATCCTGG

Gibbon TTTCCTGGTCTTGTGACAAGAACCTGGATTTTAGCTGAACTAAGGAGCAAAAGATCCTGG

Crab-eating TTTCCCGGTCTTGTGACAAGAACCTGGATTTTAGCTGAACTAAGGAGCAAAAAATCCTGG

Rhesus TTTCCCGGTCTTGTGACAAGAACCTGGATTTTAGCTGAACTAAGGAGCAAAAAATCCTGG

Marmoset TTTCCTGGCTATGTGACAAGAACGTGGATTTTAGCTGAACTAAG----------------

***** ** ************ * *****************

Human ATCATTTTGGTGACCCATACTGGTACATGAGGAAAGCTCCTATTCCAGATTCTCAGAGTA

Chimp ATCATTTTGGTGACCCATACTGGTACATGAGGAAAGCTCCTATTCCAGATTCTCAGAGTA

Gorilla ATCATTTTGGTGACCCATACTGGTACATGAGGAAAGCTCCTATTCCAGATTCTCAGAGTA

Orangutan ATCATTTTGGTGGCCCATACTGGTACATGAGGAAAGCTCCTATTCCAGATTCTCAGAGTA

Gibbon ATCATTTTGGTGGCCCATATTGGTACATGAGGAAAGCTCCTATTCCAGATTCTCAGAGTA

Crab-eating ATCATTTTGGTGGCCCATACTGGTACATGAGGAAAGCTCCTATTCCAGATTCTCAGAGTG

Rhesus ATCATTTTGGTGGCCCATACTGGTACATGAGGAAAGCTCCTATTCCAGATTCTCAGAGTG

Marmoset -------------------------------------TCCTATTCCAAATTCTCAGAATA

********** ********* *

Human TAGATTCTGACTGGATTACCCTGGTCCTTGAAGTATACCTAGGAGATGGAATAACATTGT

Chimp TAGATTCTGACTGGATTACCCTGGTCCTTGAAGTATACCTAGGAGATGGAATAACATTGT

Gorilla TAGATTCTGACTGGATTACCCTGGTCCTTGAAGTATACCTAGGAGATGGAATAACATTGT

Orangutan TAGATTCTGACTGGATTACCCTGGTCCTTGAAGTATACCTAGGAGATGGAATAACATTGT

Gibbon TAGATTCTGACTGGATTACCCTGGTCCTTGAAGTATACCTAGGAGATGGAATAACATTGT

Crab-eating TAGATCCTGACTGGATTACCCTGGTCCTTGAAGTATACCTAGGAGATGGAATAACATTGT

Rhesus TAGATCCTGACTGGATTACCCTGGTCCTTGAAGTATACCTAGGAGATGGAATAACATTGT

Marmoset TAGCTTCTGACTGGATTACC----------AAGTATGTTTAGGAGATGGAATAACATTGA

*** * ************** ****** ********************

Human ATAAGCATCCACGTTGTCATTAAGAGGAAAGAGTTTCCATTAAAACAGCAGGTCACAAAA

Chimp ATAAGCATCCATGTTGTCATTAAGAGGAAAGAGTTTCCATTAAAACAGCAGGTCACAAAA

Gorilla ATAGGCATCCATGTTGTCATTAAGAGGAAAGAGTTTCCATTAAAACAGCAGGTCATAAAA

Orangutan ATAAGCATCCATGTTGTCATTAAGAGGAAAGAGTTTCCATTAAAACAGCAGGTCATAAAA

Gibbon ATAAGCATCCATGTTGTCATTAAGAGGAAAGAGTTTCCATTAAAACAG------------

Crab-eating ACAAGCATCCATATTGCCATTAAGAGGAAAGAGTTTCCATTAAAA-AGCAGGTCATAAAA

Rhesus ACAAGCATCCATATTGCCATTAAGAGGAAAGAGTTTCCATTAAAA-AGCAGGTCATAAAA

Marmoset ACATGCATCCATGTTGTCATTAAGAGGAAAGAGTTTCCATTAAAA---------------

* * ******* *** ****************************

Human CCCTCCTGTGAGAGGAACCCTCTGTGGAAGAACAGAATATGTCACCCCAAAATATGAAGA

Chimp CCCTCCTGTGAGAGGAACCCTCTGTGGAAGAACAGAATATGTCACCCCAAAATATGAAGA

Gorilla CCTTCCTGTGAGAGGAACCCTCTGTGGAAGAACAGAATATGTCACCCCAAAATATGAAGA

Orangutan CCCTCCTGTGAGAGGAACCCTCTGTGGAAGAACAGAATATGTCACCCCAAAATATGAAGA

Gibbon ------------------------------------------------------------

Crab-eating CCCTCCTGTGAGAGGAACCCTCTGTGGAAGAACAGAATATGTCACCCCAAAATACGAAGA

Rhesus CCCTCCTGTGAGAGGAACCCTCTGTGGAAGAACAGAATATGTCACCCCAAAATACGAAGA

Marmoset ------------------------------------------------------------

Human ATTGTTGAGCTGAAGATGATTAAGAAGAAGCGGATGCAGGAAAGCTCTCTGCTCTCCCTC

Chimp ATTGTTGAGCTGAAGATGATTAAGAAGAAGCGGATGCAGGAAAGCTCTCTGCTTTCCCTC

Gorilla ATTGTTGAGCTGAAGATGATTAAGAAGAAGCGGATGCAGGAAAGCTCTCTGCTCTCCCTC

Orangutan ATTGTTGAGCTGAAGATGATTAAGAAGAAGCAGATGCAGGAAAGCTCTCTGCCCTCCCTC

Gibbon ------------------------------------------------------------

Crab-eating ATTGTTGAGCTGAAGATGATTAAGAAGAAGCAGATGCAGGAAAGCTCTCTGCCCTCCCTC

Rhesus ATTGTTGAGCTGAAGATGATTAAGAAGAAGCAGATGCAGGAAAGCTCTCTGCCCTCCCTC

Marmoset ------------------------------------------------------------

Human TATTTGCTTAAAAGCAGGATAAACACTTACAAAGACTAAAGACGCCACAATCAAGAACTC

Chimp TATTTGCTTAAAAGCAGGATAAACACTTACAAAGACTAAAGACGCCACAATCAAGAACTC

Gorilla TATTTGCTTAAAAGCAGGATAAACACTTACAAAGACTAAAGACGCCACAATCAGGAACTC

Orangutan TATTTGCTTAAAAGCAGGATAAAGACTTACAAAGACTAAAG--GCCACAATCAAGAACTC

Gibbon ------------------------------------------------------------

Crab-eating TATTTGCTTAAAGGCAGGATAAAGACTTACAAAGACTAAAG-------------------

Rhesus TATTTGCTTAAAGGCAGGATAAAGACTTACAAAGACTAAAG-------------------

Marmoset ------------------------------------------------------------

Human AGAAGGGTAGAAGAAAAAGATAGTTTCCCTTCCCTACACAGGTGTTGATCCCTTCATAAA

Chimp AGAAGGGTAGAAGAAAAAGATAGTTTCCCTTCCCTACACAGGTGTTGATCCCTTCATAAA

Gorilla AGAAGGGTAGAAGAAAAAGATAGTTTTCCTTCCCTACACAGGTGTTGATCCCTTCATAAA

Orangutan AGAAGGGTAGAAGGAAAAGATAGTTTTCCTTCCCTACACAGGTGTTGATCCCTTCATAAA

Gibbon ------------------------------------CACAGGTGTTGATCCCTTCATAAA

Crab-eating ---------GAAGAAAAAG-----------------CACAGGTGTTGATCCCTTCATAAA

Rhesus ---------GAAGAAAAAG-----------------CACAGGTGTTGATCCCTTCATAAA

Marmoset ------------------------------------------------------------

Human TATTATGCATGCTAAGCCCTATCTCAAGGTCTACATCCAGGAGAACCCAACCTGCAACAT

Chimp TATTATGCATGCTAAGCCCTATCTCAAGGTCTACATCCAGGAGAACCCAACCTGCAACAT

Gorilla TATTATGCATGCTAAGCCCTATCTCAAGGTCTACATCCAGGAGAACCCAACCTGCAACAT

Orangutan TATTATGCATGCTAAGCTCTATCTCAAGGTCTGCACCCAGGAGAACCCAACCTGCAACGT

Gibbon TATTATGCATGCTAAGCTCCATCTCAAGGTCTACATCCAGGAGAACCCAACTTGCAACAT

Crab-eating TATTATGCATGCCAAGTTCTCTCTCAAGGTCTATATCAAGGAGAACCCAACCTGCAACAT

Rhesus TATTATGCATGCCAAGTTCTCTCTCAAGGTCTATATCAAGGAGAACCCAACCTGCAACAT

Marmoset ------------------------------------------------------------

Human TAAGTTACTGACTGTGCAAGAACCCTTTGATCACGTACCTTACAGACCAAGATAAGTGAG

Chimp TAAGTTACTGACTGTGCAAGAACCCTTTGATCATGTACCTTACAGACCAAGATAAGTGAG

Gorilla TAAGTTACTGACTGTGCAAGAACCCTTTGATCATGTACCTTACAGACCAAGATAAGTGAG

Orangutan TAAGTTACTGACTGTGCAAGAACCCTTTGATCATGTACCTTACAGACCAAAATAAGTGAG

Gibbon TAAGTTACTGATTGTGCAAGAACCCTTTGATCATGTACCTTACAGACTAAGGTAAGCGAG

Crab-eating TAAGTTACTGACTGTGCAAGAACCCTTTGATCATGTACCTTACAGACCAAGGTAAGTGAG

Rhesus TAAGTTACTGACTGTGCAAGAACCCTTTGATCATGTACCTTACAGACCAAGGTAAGTGAG

Marmoset ------------------------------------------------------------

Human GATGCCCATGTATA----ACCTGCTGTCTTTCTGGTCTCTT--ATGTTTTTGTACCAGAA

Chimp GATGCCCATGTATA----ACCTGCTGTCTTTCTGGTCTCTT--ATGTTTTTGTACCAGAA

Gorilla GATGCCCATGTAAA----ACCAGCTGTCTTTCTGGTCTCTT--ATGTTTTTGTACCAGAA

Orangutan GATGCCCATGTATA----ACCTGCTGTCTTTCTGGTTTCTT--ATGTTTTTGTACCAGAA

Gibbon AAAGCCCATGTATA--TAACCTGCTGTCTTTCTGGTCTCTT--ATGTGTTTGGACCAGAA

Crab-eating GATGCCCATGTATAACCAACCTGCTGTCTTTCTGGTCTCTT--A--TTTTTTTACCAGAA

Rhesus GATGCCCATGTATAACCAACCTGCTGTCTTTCTGGTCTCTTACATTTTTTTTTACCAGAA

Marmoset ------------------------------------------------------------

Human AGGGACAACTTTTTGGGTAACCATAGGTCTCTAGTATATCTGTGATTACAATAAAGATTT

Chimp AGGGACAACTTTTTGGGTAACCATAGGTCTCTAGTATATCTGTGATTACAATAAAGATTT

Gorilla AGGGACAACTTTTGGGGTAACCATAGGTCTCTAGTATATCTGTGATTACAATAAAGATTT

Orangutan AGGGACAACTTTTTGGGTAACCATAGGTCTCTAGTATATCTGTGATTACAATAAAGATTT

Gibbon AGGGACAACTTTTTGGGTAACCATAGGTCTCTAGTATATCTGTGATTACAATAAAGATTT

Crab-eating AGGGACAACTTTTTGGGTAACCATAGGTCTCCAGTATATCTGTGATTACAATAAAGATTT

Rhesus AGGGACAACTTTTTGGGTAACCACAGGTCTCCAGTATATCTGTGATTACAATAAAGATTT

Marmoset ------------------------------------------------------------

Human TTGATATACCAAAACTGATGAACCAAAATTCCACTGAAATAGCGAGTAAATGGAGTTCTT

Chimp TCGATATACCAAAACTGATGAACCAAAATTCCACTGAAATAGCGAGTAAATGGAGTTCTT

Gorilla TTGATATACCAAAACTGATGAACCAAAATTCCACTGAAATAGCGAGTAAATGGAGTTCTT

Orangutan TTGATATACCAAAACTGATGAACCAAAATTCCACTGAAATAGCGAGTGAATGGAGTTCTT

Gibbon TTGATATACCAAAACTGATGAACCAAAATTCCACTGAAATAGCAAGTAAATGGAGTTCTT

Crab-eating TTGATATACCAAAACTGATGAACCTAAATTCCACTGAAATAGAGAGTAAATGGAGTTATT

Rhesus TTGATATACCAAAACTGATGAACCTAAATTCCACTGAAATAGAGAGTAAATGGAGTTATT

Marmoset ------------------------------------------------------------

Human TTATCCTTTGCAGTATACCAGGGTGAACTTTTTACATGATTTCTTGCTCCAAAGGGAGAA

Chimp TTATCCTTTGCAGTATACCAGGGTGAACTTTTAACATGATTTCTTGCTCCAAAGGGAGAA

Gorilla TTATCCTTTGCAGTATACCAGGGTGAACTTTTTACATAATTTCTTGCTCCAAAGGGAGAA

Orangutan TTATCCTTTGCAGTATACCAGGGTGAACTTTTTACATGATTTCTTGCTCCAAAGGGAGAG

Gibbon TTATCCTTTGCAGTATATTAGGGTGAACTTTTTACATGATTTCTTGCTCCAAAGGGAGAG

Crab-eating TTAACCTTTGCAGTATACCAGGGTGAACTTTTTACATGATTTCTTGCTCCAAAGGGAGAG

Rhesus TTAACCTTTGCAGTATACCAGGGTGAACTTTTTACATGATTTCTTGCTCCAAAGGGAGAG

Marmoset ------------------------------------------------------------

Human GTGTTATGACAAAGTACATTTAAGGACCCTAACTGATCACAGAAAAAATATTAATGGCAT

Chimp GTG-TATGACAAAGTACGTTTAAGGACCCTAACTGATCATAGAAAAAATATTAATGGCAT

Gorilla GTG-TATGACAAAGTACGTTTAAGGACCCTAACTGATCATAGAAAAAATATTAATGGCAT

Orangutan GTG-TATGACAAAGTACGTTTAAGGACCCTAACTGATCATAGAAAAAATATTAATGGCAT

Gibbon GTG-TATGACAAAGTATGTTTAAGGACCCTAACTGGTCATAGAAAAAATATTAATG--AT

Crab-eating GTG-TATGACAAAGTACTTTTAAAGACTCTAACTGATCATAGAAAAACTATTAATGGCAT

Rhesus GTG-TATGACAAAGTACTTTTAAAGACTCTAACTGATCATAGAAAAAATATTAATGGCAT

Marmoset -------GACAAAGTACTTTTAAAGACTCTAACTGATTACAGAAAAAATATTAATGACAT

********* ***** *** ******* * * ******* ******** **

Human TGGGAAAAAAATGCAGACTAATGAATCCACTACTAAATTTTATCTATCTTGTAGCTCTAA

Chimp TGGGAAAAAAATGCAGACTAATGAATCCACTACTAGATTTTACCTATCTTGTAGCTCTAA

Gorilla TGGGAAAAAAATGCAGACTAATGAATCCACTACTAAATTTTATCTATCTTGTAGCTCTAA

Orangutan TGGGAAAAAAATGCAGACTAATGAATCCACTACTAAATTTTATCTATCTTGTAGCTCTAA

Gibbon TGGGAAAAAAATGCAGACTAATGAATCCACTACTAAACTTTATCTATCTTGTAGCTCTAA

Crab-eating TGGGA-AAAAATGCAGACTAATGAATCCACTACTAAATTTTATCTATCTTGTAGCTCTTA

Rhesus TGGGA-AAAAATGCAGACTAATGAATCCACTACTAAATTTTATCTATCTTGTAGCTCTTA

Marmoset TGGGGCAAAAATCCAGACTAATGAATCCACTACTAGATTTTATCTATCTTGTAGCTCTAA

**** ****** ********************** * **** *************** *

Human AATTGCACTGTTTAGAATACACTATGGTAGCCACTAGACATATGGCTACTTGAACTTGAA

Chimp AATTGCACTGTTTAGAATACACTATGGTAGCCACTAGACATATGGCTACTTGAACTTGAA

Gorilla AATTGCACTGTTTAGAATACACTATGGTAGCCACTAGACATATGGCTACTTGAACTTGAA

Orangutan AATTGCACTGTTTAGAATACACTATGGTAGCCACTAGACATATGGCTACTTGAACTTGAA

Gibbon AATTGCACTGTTTAGAATACACTGTGGTAGCCACTAGACATATGGCTACTTGAACTTGAA

Crab-eating AATTGCACTGTTTAGAATACACTATGATAGCCGCTAAACATATGGCTACTTGAACTTGAA

Rhesus AATTGCACTGTTTAGAATACACTATGATAGCCGCTAAACATATGGCTACTTGAACTTGAA

Marmoset AATTGCACTGTTTAGTATACACTGTGGTAGCCACTAGACATAGGGCTACTTGAACTTGAA

*************** ******* ** ***** *** ***** *****************

Human TTGGTTAAAATCAAAATTAAGAATTTAATTTCTCACTCACATTAGCCACATTTCAAGTGC

Chimp TTGGTGAAAATCAAAATTAAGAATTTAATTTCTCACTCACATTAGCCACATTTCAAGTGC

Gorilla TTGGTTAAAATCAAAATTAAGAATTTAATTTCTCACTCACATTAGCCACGTTTCAAGTGC

Orangutan TTGGTTAAAATCAAAATTAAGAATTTAATTTCTCACTCACATTAGCCACATTTCAAGTGC

Gibbon TTGGTTAAAATCAAAATTAAGAATTTACTTTCTCACTCACATTAGCCACATTTCAAGTGC

Crab-eating TTGGTTAAAATCAAAATTAAGAATTTAATTTCTCACTCACATTAGCCACATTTCACGTGC

Rhesus TTGGTTAAAATCAAAATTAAGAATTTAATTTCTCACTCACATTAGCCACATTTCACGTGC

Marmoset TTACTTAATATCAAAATTAAGACTTTAATTTCTCATTCACATTAGCCACAATTTAAGTGA

** * ** ************* **** ******* ************* ** * ***

Human TCAACAGACACATGTGGCTAGTGTCTACCATATTGGACAGCATAGATTTAAAGCACATTT

Chimp TCAACAGACACATGTGGCTAGTGTCTACCATATTGGACAGCATAGATTTAAAGCACATTT

Gorilla TCAACAGACACATGTGGCTAGTGTCTACCATATTGGACAGCATAGATTTAAAGCACATTT

Orangutan TCAACAGACACATATGGCTAGTGTCTACCATATTGGATAGCATAGATTTAAAGCACATTT

Gibbon TCAACAGACACATGTGGCTAGTGTCTACCATATTGGACAGCATAGACTTAAAGCACATTT

Crab-eating TCAACAGACACATGTGACTAGTGTCTACCATATTGGACAGCATAGATTTAAAGAACATTT

Rhesus TCAACAGACACATGTGACTGGTGTCTACCATATTGGACAGCATAGATTTAAAGAACATTT

Marmoset TCAAAAGACACATGTGGCTAGTGCCTACCATATTGCACAGCATAGATCTAAAGAACATTT

**** ******** ** ** *** *********** * ******** ***** ******

Human CCATCATTGCAACACTGCAATATGGAGGATGTATCCTCAATACATTGATATTGCCCCCTC

Chimp CCATCATCGCAACACTGCAATATGGAGGATGTATCCTCAATACACTGATATTGCCCCCTC

Gorilla CCATCATTGCAACACCGCAATATGGAGGATGTATCCTCAATACATGGATATTGCCCCCTC

Orangutan CCATCATGGCAACATTGCAATATGGAGGATGTATCCTCAATACATTGATATTGCTCCCTC

Gibbon CCATCATTGCAACATTGCAATATGGAGGATGTATCCTCAATACATTGATATTGCCCCCTC

Crab-eating CCATCGTTGCAACATTGCAATTTTGAGGATGCATCCTCAATACATTGATATTGCCCCCTC

Rhesus CCATCGTTGCAACATTGCAATTTTGAGGATGCATCCTCAATACATTGATATTGCCCCCTC

Marmoset CCATCACTGCAACA----------GAGGATACATCCTCAGTACACTGATATTGTCCCCTC

***** ****** ****** ******* **** ******* *****

Human AAAATTCATAACCCCTAGATATCTTATCTCTGTATGTGAGGATAGTGTATTAACTTGCAC

Chimp AAAATTCATAACCCCTAGATATCTTATCTCTCTATGTGAGGATAGTGTATTAACTTGCAC

Gorilla AAAATTCATAACCCCTAGATATCTTATCTCTCTATGTGAGGATAGTGTATTAACTTGCAC

Orangutan AAAATTCATAACCCCTAGATATCTTATCTCTCTATGTGAGGATAGTGTATTAACTTGCAC

Gibbon AAAATTCATAACCCCTAGCTATCTTATCTCTCTATGTGAGGATAGTATGTTAGCTTGCGC

Crab-eating AAAATTCATAACCCCTAGATATCTTATCTCTCTATGTGAGGATAGTGTATTAACTTACAC

Rhesus AAAATTCATAACCCCTAGATATCTTATCTCTCTATGTGAGGATAGTGTATTAACTTACAC

Marmoset AAAATTCATAACACTTAGATATCTTATC----TATGTGAGGATAGTGTATCAACTTATAC

************ * *** ********* ************** * * * *** *

Human CTGGACTGAGATAGATAAATGCTTATCTCCAGAGATGTCTGAAAGTCTGGTACTGACCCT

Chimp CTGGACTGAGATAGATAAATGCTTATCTCCAAAGATGTCTGTAAGTCTGGTACTGACCCT

Gorilla CTGGACTGAGATAGATAAATGCTTATCTCCAGAGATGTCTGAAAGTCTGGTACTGACCCT

Orangutan CTGGACTGAGATAGATAAATGCTTATCTCCAGAGATGTCTGAAAGTCTAGTACTGACCCT

Gibbon CTGGACTGAGATAGATAAATGCTTATCTCCAGAGATGTCTGAAAGTCTGGTACTGACCCT

Crab-eating CTGGACTGAGATAGATAAATGCTTATCTCCAGAGATGTCTGAAAGTCTGGTACTGATCCT

Rhesus CTGGACTGAGATAGATAAATGCTTATCTCCAGAGATGTCTGAAAGTCTGGTACTGATCCT

Marmoset CTGGACTGGGATAGATAAATGCTTATCTCCAGAGATGTCTGAAAGTCTGGTACTGACCCT

******** ********************** ********* ****** ******* ***

Human TGTCTAAGATAAGGATGGAATGTGCTGGTTAATGATTTATACTGTGCTATACAAAGTCCA

Chimp TGTCTAAGATAAGGATGGAATGTGCTGGTTAATGATTTATACTGTGCTATACAAAGTCCA

Gorilla TGTCTAAGATAAGGATGGAATGTGCTGGTTAATGATTTATACTGTGCTATACAAAGTCCA

Orangutan TGTCTAAGATAAGGATGGAATGTGCTGGTTAATGATTTATACTGTGCTATACAAAGTCCA

Gibbon TGTCTAAGATAAGGATGGAATGTGCTGGTTAATGATTTATACTGTGCTATACAAAGTCCA

Crab-eating TGTCTAACATAAGGATGGAATGTGCTGGTTAATGATTTATACTGTGCTATGCAAAGTTCA

Rhesus TGTCTAACATAAGGATGGAATGTGCTGGTTAATGATTTATACTGTGCTATGCAAAGTTCA

Marmoset TGTCTAAGATAAAGATGGAATGTACTGGTTAATGATTTATACTGTGCTATTCAAAGACCA

******* **** ********** ************************** ***** **

Human AAATTCCTTTGGAGGTATCCTCTGCTGGCTTGTCTACATTGTTTAAGGATGGATAGGTTG

Chimp AAATTCCCTTGGAGGTATCCTCTGCTGGCTTGTCTACATTGTTTAAGGATGGATAGATTG

Gorilla AAATTCCCTTGGTGGTATCCTCTGCTGGCTTGTCTACATTGTTTAAGGATGGATAGATTG

Orangutan AAATTCCCTTGGAGGTATCCTCTGCTGGCTTGTCTACATTGTTTAAGGATGGATAGATTG

Gibbon CAATTCCCTTGGAGGTATCCTCTGCTGGCTTGTCTACATTGTTTAAGGATGGATAGATTT

Crab-eating AAATTCCCTTGGAGGTGTCTTCTGCTGGCTTGTCTACATTGTTTAAGAATGTATAGATTG

Rhesus AAATTCCCTTGGAGGTGTCTTCTGCTGGCTTGTCTACATTGTTTAAGAATGTATAGATTG

Marmoset TAATTCCTTTGGACGTATCCTGTGTTGGCTTGTCTACATTGTTTAAGGATTTATAGATTG

****** **** ** ** * ** ********************** ** **** **

Human ATGGTTTGCACCTGCTTTAATCATTTACACATTCTGATTTTATTTGTGATCAGTGGAGGA

Chimp ATGGTTTGCACCTGCTTTAATCATTTACGCATTCTGATTTTATTTGTGATCAGTGGAGGA

Gorilla ATGGTTTGCACCTGCTTTAATCATTTACACATTCTGATTTTATTTGTGATCAGTGGAGGA

Orangutan ATGGTTTGCACCTGCTTTAATCATTTACACATTCTGATTTTATTTGTGATCAGTGGAGGA

Gibbon ATGGTTTGCTCCTGCTTTAATCATTTACACATTCTGATTTTATTTGTGATCAGTGGAGGA

Crab-eating ATGGTTTGCACCTGCTTTAATCATTTACACATTCTGATTTTATTTGTGATCAGTGGAGGA

Rhesus ATGGTTTGCACCTGCTTTAATCATTTACACATTCTGATTTTATTTGTGATCAGTGGAGGA

Marmoset ATGGTTTCCACCTGGTTTAATCATTTACACATTCTGATTTTATTTGTGATCA------GA

******* * **** ************* *********************** **

Human TAAAACCTTCAATAAACTTGTGTCTTAGCTTCCTTGATATTAAGATATCTTGCACATATC

Chimp TAAAACCTTCAATAAACTTGTGTCTTAGCTTCCTTGATATTAAGATATCTTGCACATATC

Gorilla TAAAACCTTCAATAAACTTGTGTCTTAGCTTCCTTGATATTAAGATATCTTGCACATATC

Orangutan TAAAACCTTCAATAAACTTCTGTCTTAGCTTCCTTGATATTAAGATATCTTTCACATATC

Gibbon TAAAACCTTCAATAAACTTGTGTCTTAGCTTCCTTGATATTAAGAGATCTTGCACATATC

Crab-eating TAAAACCTTCAATAAACTTGTGTCTTAGCTTCCTTGATATTAAGATATCTTGCACATATC

Rhesus TAAAACCTTCAATAAACTTGTGTCTTAGCTTCCTTGATATTAAGATATCTTGCACATATC

Marmoset T-TTATCTTCAGTAAACTTGTGTCTTAGCTTACTTGATATTAAGATATCTTGCACATATC

* * ***** ******* *********** ************* ***** ********

Human TTAGATTTTTATAATCTGAATGCAAAGCACATCCTTTGCAATTGAGAAAGGACTCTGTGA

Chimp TTAGATTTTTATAATCTGAATGCAAAGCACATCCTTTGCAATTGAGAAAGGACTCTGTGA

Gorilla TTAGATTTTTATCATCCGAATGCAAAGCACATCCTTTGCAATTGAGAAAGGACTCTGCGA

Orangutan TTAGTTTTTTATAATCTGAATGCAAAGCACATCCTTTGCAATTGAGAAAGGACTCTGTGA

Gibbon TTCGTTTTTTATAATCTGAATGCAAAGCACATCCTTTGCAATTGAGAAAGGACTCTGTGA

Crab-eating TTAGTTTTTTATAATCTGGATGCAAAGCACATCCCTTGCAATTGATAAAGGACTCTGTGA

Rhesus TTAGTTTTTTATAATCTGGATGCAAAGCACATCCTTTGCAATTGATAAAGGACTCTGTGA

Marmoset T----TTTTCATAATCTGAATGCAAATCACATCCTTTGCAACTGAGAAAGGACTCTGAGA

* **** ** *** * ******* ******* ****** *** *********** **

Human GCTGCTCCTGGATATGTGAAACCCCTCAGTGACTGACCGTATTTTCTTTCTTCTGCTGTA

Chimp GCTGCTCCTGGATATGTTAAACCCCTCAGTGACTGACCGTGTTTTCTTTCTTCTGCTGTA

Gorilla GCTGCTCCCGGATATGTTAAACCCCTCAGTGACTGACTGTATATTCTTTCTTCTGCTGTA

Orangutan GCTGCTCCTGGACATGTTAAACCCCTCAGTGACTGACCGTATTTTCTTTCTTCTGCTATA

Gibbon GCTGCTCCTGGACATGTTAAACCCCTCAATGACGGACCGTATTTTCTTTCTTCTGCTATA

Crab-eating GCTGCTCCTGGACATGTTAAACCCCTCAATGACTCACTGTATTTTCTTTCTTCTGCTATA

Rhesus GCTGCTCCTGGACATGTTAAACCCCTCAATGACTCACTGTATTTTCTTTCTTCTGCTATA

Marmoset GCTGCTCCTGGACATGTTAAACCCCTCAATGACTGACTGTATTTTCTTTCTTCTGCTATA

******** *** **** ********** **** ** ** * ************** **

Human CTTGCCCTTATTAAAGCCTTACATGTGAAGTAAGTCCTTTCAATTGTCTGACTCCGGGTG

Chimp CTTGCCCTTATTAAAGCCTTACATGTGAAGTAAGTCCTTTCAATTGTCTGACTCCGGGTG

Gorilla CTTGCCCTTATTAAAGCCTTACATGTGAAGTAAGTCCTTTCAATTGTCTGACCCCGGGTG

Orangutan CTTGCCCTTATTAAAGCCTTATACGTGAAGTAAGTCCTTTCAATTGTCTGACCCTGGGTG

Gibbon CTTGCCCTTATTAAAGCCTTATATGTGAAATAAGTCCTTTCAATTGTCTGACCCTGGGTG

Crab-eating CTTGCCCTTATTAAAGCCTTACATGTGAAGTAAGTCATTTCAATTGTCTGACCCTGGGTG

Rhesus CTTGCCCTTATTAAAGCCTTACATGTGAAGTAAGTCATTTCAATTGTCTGACCCTGGGTG

Marmoset CTTGCCTTTATTAAAGCCTTACATGTGAAGTAAGTTCTTTCAATTGTCTGACCCTGGGTG

****** ************** * ***** ***** *************** * *****

Human ATTGTTATACCCTTTGATATAGTTTGGATATTTGTGCCTTCCAAATCTCATGTTGAAATG

Chimp ATTGTTATACCCTTTGATATAGTTTGGATATTTGTGCCTTCCAAATCTCATGTTGAAATG

Gorilla ATTGTTATACCCTTTGATATAGTTTGGATATTTGTGCCTTCCAAATCTCATGTTGAAATG

Orangutan ATTGTTATACCCTTTGATATAGTTTGGATATTTGTGCCTTCCAAATCTCATGTTGAAATG

Gibbon ATTGTTATACCCTTTGATATAGTTTGGATATTTGTGCCTTCCAAATCTCATGTTGAAATG

Crab-eating ATTGTTATACCCTTTGATGTAGTTTGGATATTTGTACCTTCCAAATCTCATGTTGAAATG

Rhesus ATTGTTATACCCTTTGATGTAGTTTGGATATTTGTACCTTCCAAATCTCATGTTGAAATG

Marmoset ATTGTTATACCCTTTGATATAGTCTGCATATCTGTGCCTTTCAAATATCATGTTGAAATG

****************** **** ** **** *** **** ***** *************

Human CGATCCCCTATGTTGGAGGTGGAGCCTAGTAGGAGGTGTTAGGGTCACGGGAATGGATCC

Chimp TGATCCCCTATGTTGGAGGTGGAGCCTAGTAGGAGGTGTTAGGGTCACGGGAATGGATCC

Gorilla TGATCCCCTATGTTGGAGGTGGAGCCTAGTAGGAGGTGTTAGGGTCACGGGAATGGATCC

Orangutan TGATCCCCCATGTTGGAGGTGGAGCCTAGTAGGAGGTGTTAGGGTCACAGGAATGGATCC

Gibbon TGATCCCCCATGTTGGAGGTGGAGCCTAGTAGGAGGTGTTAGGGTCACGGGAATGGATCC

Crab-eating TGATCCCCCAAGTTGGAGGTGGAGCCTAGTGGGAGGTGTTAGGGTTGTGGGAATGGATCC

Rhesus TGATCCCCCAAGTTGGAGGTGGAGCCTAGTGGGAGGTGTTAGGGTTGTGGGAATGGATCC

Marmoset TGATCCCTCATGTTGGAGGTGGAGCCTAGTGGGAGGTATTAGGGTCATGAGGATGGATCC

****** * ******************* ****** ******* * ********

Human CTGATGAATGGCTTGGTACACAGTAATGAGTGAGTTTGTGCTTTATTAGTTACTGTGAGA

Chimp CTGATGAATGGCTTAGTACACAGTAATGAGTGAGTTTGTGCTTTATTAGTTACTGTGAGA

Gorilla CTGATGAATGGCTTGGCACACAGTAATGAGTGAGTTTGTGCTTTATTAGTTACTGTGAGA

Orangutan CTGATGAATGGCTTGGTACACAGTAATGAGTGAGTTTGTGCTTTATTAGTTACTGTGAGA

Gibbon CTGATGAATGGCTTGGTACACAGTAATGAGTGAGTTTGTGCTTTATTAGTTACTGTGAGA

Crab-eating CTGATGAATGGCTTGGTACACAGTAATGAGTGAGTTTGTGCTTTATTAGTTACTGTGAGA

Rhesus CTGATGAATGGCTTGGTACACAGTAATGAGTGAGTTTGTGCTTTATTAGTTACTGTGAGA

Marmoset CTGATGAATAGCTTGGTGCACAGTAATAAGTGAGTTTGTGCTTTATTAGTTACTGTGAGA

********* **** * ********* ********************************

Human TTTACTTGGTAAAAAGAGCCTGGCAACTCCT-CCCTCTCCCTTGCTCCCTCTCTTGCCAT

Chimp TTTACTTGGTGAAAAGAGCCTGGCAACTCCT-CCCTCTCCCTTGCTCCCTCTCTTGCCAT

Gorilla TTTACTTGGTAAAAAGAGCCTGGCAACTCCT-CCCTCTCCCTTGCTCCCTCTCTTGCCGT

Orangutan TTTACTTGGTAAAAAGAGCCTGACAACTCCTCCCCTCTCCCTTGCTCCCTCTCTTGCCAT

Gibbon TTTACTTGGTAAAAAGAGCCTGGCAACTCCTCCCCTCTCCCTTGCTCCCTCTCTTGCCAT

Crab-eating TCTGCTTGGTAAAAAGATCCTGACATCTCCTCCCCTCTCCCTTGCTCCCTCTCTCGCCAT

Rhesus TCTGCTTGGTAAAAAGATCCTGACATCTC---CCCTCTCCCTTGCTCCCTCTCTCGCCGT

Marmoset TCTGCTTGGTAAAAAGAGCCTGGCATCTACTCCCCTCTCTCTTGCTCCCTCTCTTGCCAT

* * ****** ****** **** ** ** ******* ************** *** *

Human GTGACATGCTTGCTCCAGCTGCACCTTTTGCCACAATTGTAAGCTTTGTGAGTCCCTCAA

Chimp GTGACATGCTTGCTCCAGCTGCACCTTTTGCCACAATTGAAAGCTTTGTGAGTCCCTCAA

Gorilla GTGACATGCTTGCTCCAGCTACACCTTTTGCCACAATTGTAAGCTTTGTGAGTCCCTCAA

Orangutan GTGACATGCTTGCTCCAGCTGCACCTTTTGCCACAATTTTAAGCGTTGTGAGTCCCTCAA

Gibbon GTGACATGCTTGCTCCAGCTGCACCTTTTGCCACAATTGTAAGCTTTGTGAGTCCCTCAA

Crab-eating GTGACACGCTTGCTCCAGCTGCACCTTTTGCCACAGTTGTAAGCTTTGTGAGTCCCTCAA

Rhesus GTGACATGCTTGCTCCAGCTGCACCTTTTGCCACAGTTGTAAGCTTTGTGAGTCCCTCAA

Marmoset GTGAAATGCTTGCCCTGGCTTCATCTTCCACTACAATTGTAAGCTTTGTGAGTCTCTCAT

**** * ****** * *** ** *** * *** ** **** ********* ****

Human CAGAAGCTGAGCAAATGCTGGCACCATGCTTCTTGTACAGCCT-----ACAGAACTGTGA

Chimp CAGAAGCTGAGCAAATGCTGGCACCATGCTTCTTGTACAGCCT-----ACAGAACTGTGA

Gorilla CAGAAGCTGAGCAAATGCTGGCACCATGCTTCTTGTACAGCCT-----ACAGATCTGTGA

Orangutan CAGAAGCTGAGCAAATGCTGGCACCGTGCTTCTTGTACAGCCT-----ACAGAACTGTGA

Gibbon CAGAAGCTGAGCAAATGCTGGCACCAAGCTTCTTGTACAGCCT-----ACAGAACTGTGA

Crab-eating CAGAAGCTGAGCAAATGCTGGCACCATGCTTCTTGTACAGCCTACAGAACAGAACTGTGA

Rhesus CAGAAGCTGAGCAAATGCTGGCACCATGCTTCTTGTACAGCCTACAGAACAGAACTGTGA

Marmoset CAGAAGCTGAGCAGATGCTGGCACCACATTTCTTGTTCAGCCT-----ATAGGACTGTGA

************* *********** ******* ****** * ** ******

Human ACCAAATAAAACTTTTATTTTTAT

Chimp ACCAAATAAAACTTTTATTTTTAT

Gorilla ACCAAATAAACGTTTTATTTTTAT

Orangutan ACCAAATAAACCTTTTATTTTTAT

Gibbon ACCAAATAAACCTTTTATTTTTAT

Crab-eating ACCAAATAAACCTTTTATTTTTAT

Rhesus ACCAAATAAACCTTTTATTTTTAT

Marmoset ACCAAATAAACCGTTTCTCTTTAT

********** *** * *****

**Figure 1 - figure supplement 6 - source data 1.** Multiple alignment of complete sequence of human *PINCR* mature transcript is presented for different primate orthologous sequences. The alignment was built by the Muscle program with default parameters. Some genome rearrangements were found in Rhesus, Crab-eating macaque, and Marmosets orthologous sequences, and parts of the sequences conserved to human *PINCR* mature transcript are transcribed from the reverse strands of their genomes (shown in yellow), duplicate parts that are present in both strands (+ and -) are shown in green. Genome rearrangements were analyzed using the Owen program for pair-wise alignments.
