# Supplementary material for: Prosurvival long noncoding RNA PINCR regulates a subset of p53 targets in human colorectal cancer cells by binding to Matrin 3
Source: eLife. 2017 Jun 5;6:e23244. doi: 10.7554/eLife.23244 (PMC5470874; doi:10.7554/eLife.23244)
Supplement: Figure 1—figure supplement 6—source data 2. — DOI: http://dx.doi.org/10.7554/eLife.23244.012 [file elife-23244-fig1-figsupp6-data2.docx]

**Multiple sequence alignment of *PINCR* promoter**

**Figure 1 - figure supplement 6 - source data 2**

Human TATTTTCAATCTTCAAAGGAAACTTGTCTGAAATTATGGGAAACTTCTTAGTCCTGGTGA

Chimp TATTTTCAATCTTCAAAGGAAACTTGTCTGAAATTATGGGAAACTTCTTAGTCCTGGTGA

Gorilla TATTTTCAATCTTCAAAGGAAACTTGTCTTAAATTATGGGAAACTTCTTAGTCCTGGTGA

Orangutan TATTTTCAATCTTCAAAGGAAACTTGTCTGAAATTATGGGAAACTTCTTAGTCCTGGTGA

Gibbon TATTTTCAATCTTCAAAGGAAACTTGTCTGAAATTATGGGAAACTTCTTAGTCCTGGTGA

Crab-eating TATTTTCAATCTTCAGAGGAAACTTGTCTGAAATTATGGGAAACTTCTTAGTCCTGGTGA

Rhesus TATTTTCAATCTTCAGAGGAAACTTGTCTGAAATTATGGGAAACTTCTTAGTCCTGGTGA

Marmoset TATTTTCAGTCTTCAAAGGAAACTTGTCTGAAATTATGGGAAAATTCTTAGTCCAGGTGA

******** ****** ************* ************* ********** *****

Human TAGTTGCAGGAGGCAGCTAAGGGAGGATCCCTGGAGAATCTCCCAACCACCCCACAAGTG

Chimp TAGTTGCAGGAGGCAGCTAAGGGAGGATCCCTGGAGAATCTCCCAACCACCCCACAAGTG

Gorilla TAGTTGCAGGAGGCAGCTAAGGGAGGATCCCTGGAGAATCTCCCAACCACCCCACAAATG

Orangutan TAGTTGCAGGAGGCAGCTAAGGGAGGATCCCTGGAGAATCTCCGACCCACCCCACAAGTG

Gibbon TAGTTGCAGGAGGCAGCTAAGGGAGGATCCCTGGAGAATCTCTGACCCACCCCACAAGTG

Crab-eating CAGTTGCAGGAGGCAGATAAGGGAGGATCCCTGGAGAATCTCCAACCCACCCCACAAGTA

Rhesus CAGTTGCAGGAGGCAGATAAGGGAGGATCCCTGGAGAATCTCCAACCCACCCCACAAGTA

Marmoset GAGTTGCAGGAGGCAGATAAGGGAAGATTCCTGGAGAATCTCCACCCCACCCCACAAGTG

*************** ******* *** ************* *********** *

Human TTTACATCAGATGCTTTTATGCACATGAGAGAACCTGCCCAGGCCCTTGTCTGGACATGC

Chimp TTTACATCAGATGCTTTTATGCACATGAGAGAACCTGCCCAGGCCCTTGTCTGGACATGC

Gorilla TTTACATCAGATGCTTTTATGCACATGAGAGAACCTGCCCAGGCCCTTGTCTGGACATGC

Orangutan TTTACATCAGATGCTTTTATGCACATGAGAGAACCTGCCCAGGCCCTTGTCTGGACATGC

Gibbon TTTACATCAGATGCTTTTATGCACATGAGAGAACCTGCCCAGGCCCTTGTCTGGACATGC

Crab-eating TTTACATCAGATGCTTTTATGCACATGAGAGAACCTGCCCAGGCCCTTGTCTGGACATGC

Rhesus TTTACATCAGATGCTTTTATGCACATGAGAGAACCTGCCCAGGCCCTTGTCTGGACATGC

Marmoset TTTACATCAGATGCTTTTATGCACATGAGAGAACCTGCCCAGGCCCTTGTCTGGACATGC

************************************************************

Human CCACAATGGACTGGGGGCCGGCCTGCTCACTGGAAGCATGGGGTGGAGCCACTGGGGATT

Chimp CCACAATGGACTGGGGGCCGGCCTGCTCACTGGAAGAATGGGGTGGAGCCACTGGGGATT

Gorilla CCACAATGGACTGGGGGCCGGCCTGCGCACTGGAAGAATGGGGTGGAGCCACCGGGGATT

Orangutan CCGCAATGGACTGCGGGCTGGCCTGTGCACTGGAAGAATGGGGT-GAGCCACTGGGGATT

Gibbon CCACAACGGACTGGGGGCTGGCCTGCACACTGGAAGAATGGGGTGGAGCCACTGGGGATT

Crab-eating CCACAATGGACTGGGGGCCTGCCTGCGCACTGAAAGAATGGGGTGGAGCCACTGGGGATT

Rhesus CCACAATGGACTGGGGGCCTGCCTGCGCACTGAAAGAATGGGGTGGAGCCACTGGGGATT

Marmoset CCACAATAGACTGGGGGCCTGCCTGTGCACTGGAAGAATGGGGTGGAGACCCTGGGGATT

** *** ***** **** ***** ***** *** ******* *** * * *******

Human CGTTCCTTATGCAGTGGGGAGGAGCGTGGCCTCTTCAGGTTGTGTGT---GTGGTGGCCT

Chimp CGTTCCTTATGCAGTGGGGAGGAGCGTGGCCTCTTCAGGTTGTGTGT---GTGGTGGCCT

Gorilla CGTTCCTTATGCAGTGGGGAGGAGCGTGGCCTCTTCAGGTTGTGTGT---GTGGTGGCCT

Orangutan CGTTCCTTATGCAGTGGGGAGGAGCGTGGCCTCTTCAGGTTGTGTGT---GTGGTGGCCT

Gibbon CGTTCCTTATGTAGTGGGGAGGAGCGTGGCCTCTTCAGGTTGTGTGT---GTGGTGGCCT

Crab-eating CGTGCCTTATGCAGTGGGGAGGAGCGTGGCCTCTTCAGGTTGTGTGT---GTGGTGGCCT

Rhesus CGTGCCTTATGCAGTGGGGAGGAGCGTGGCCTCTTCAGGTTGTGTGT---GTGGTGGCCT

Marmoset CATGTCTTATGCAGGTGGGAGGAACGTGACCTCTT-ATGTTGTGTGTATGGTGGTGGCCT

* * ****** ** ******* **** ****** * ********* **********

Human GGCATTCAATCTGTGAGGTGGAT-GCGTGTTGGAAGGACCCCTTTTGGTTTCTTTTTGCT

Chimp GGCATTCAATCTGTGAGGTGGAT-GCGTGTTGGAAGGACCCCTTTTGGTTTCTTTTTGCT

Gorilla GGCATTCAATCTGTGAGGTGGAC-GCATGTTGGAAGGACCCCTTTTGGTTTCTTTTTGCT

Orangutan GGCATTCAATCTGTGAGGTGGACGGCGTGTTGGAAGGACCCGTTTTGGTTTCTTTTTGCT

Gibbon GGCATTCAATCTGTGAGGTGGAT-GCGTGTTGGAAGGACTCCTTTTGGTTTCTTTTTGCT

Crab-eating GGCATTCAATCTGTGAGGTGGAA-GCCTGTTGGAAGGACCTCTTTTGGTTTCTTTTTGCT

Rhesus GGCATTCAATCTGTGAGGTGGAA-GCCTGTTGGAAGGACCTCTTTTGGTTTCTTTTTGCT

Marmoset GGTATTCAATCTGTAGGGTGGGA-GCCTGATGGAAGGACCGCTTTTGGGTT-TTCTTGCT

** *********** ***** ** ** ********* ****** ** ** *****

Human CAGAGC---TTTCTTTTAATAAATTCCGCTCTCCTCACCTTTCAATGTGTCCGTATGCCT

Chimp CAGAGC---TTTCTTTTAATAAATTCCGCTCTCCTCACCTTTCAATGTGTCCGTATGCCT

Gorilla CAGAGC---TTTCTTTTAATAAATTCCGCTCTCCTCACCTTTCAATGTGTCTGTATGCCT

Orangutan CAGAGC---TTTCTTTTAATAAATTCCGCTCTCCTCACCTTTCAATGTGTCCGTATGCCT

Gibbon CAGAGC---TTTCTTTTAATAAATTCCACTCTCCTCACCTTTCAATGTGTCTGTATACCT

Crab-eating CAGAGC---TTTCTTTTAATAAATTCCGCTCTCCTCACTTTTCAATGTGTCCATGTGCCT

Rhesus CAGAGC---TTTCTTTTAATAAATTCCGCTCTCCTCACTTTTCAATGTGTCCATGTGCCT

Marmoset CAGAGCTTTTTTTTTTTAATAAACTCAGCTCTCCTCACCTTTCAATGTGTCTGTGTGCCT

****** *** ********** ** ********** ************ * * ***

Human AATTTTTCCTGGTCCTGTGACAAGAACCT-GATTTTAGCTGAACTAAGGAGCGAAAGATC

Chimp AATTTTTCCTGGTCCTGTGACAAGAACCTGGATTTTAGCTGAACTAAGGAGCGAAAGATC

Gorilla AATTTTTCCTGGTCTTGTGACAAGAACCTGGATTTTAGCTGAACTAAGGAGCTAAAGATC

Orangutan AATTTTTCCTGGTCTTGTGACAAGAACCTGGATTTTAGCTGAACTAAAGAGCAAAAGATC

Gibbon AACTTTTCCTGGTCTTGTGACAAGAACCTGGATTTTAGCTGAACTAAGGAGCAAAAGATC

Crab-eating AATTTTTCCCGGTCTTGTGACAAGAACCTGGATTTTAGCTGAACTAAGGAGCAAAAAATC

Rhesus AATTTTTCCCGGTCTTGTGACAAGAACCTGGATTTTAGCTGAACTAAGGAGCAAAAAATC

Marmoset AATTTTTCCTGGCTATGTGACAAGAACGTGGATTTTAGCTGAACTAAG------------

** ****** ** ************ * *****************

Human CTGGATCATTTTGGTGACCCATACTGGTACATGAGGAAAG-

Chimp CTGGATCATTTTGGTGACCCATACTGGTACATGAGGAAAG-

Gorilla CTGGATCATTTTGGTGACCCATACTGGTACATGAGGAAAG-

Orangutan CTGGATCATTTTGGTGGCCCATACTGGTACATGAGGAAAG-

Gibbon CTGGATCATTTTGGTGGCCCATATTGGTACATGAGGAAAGG

Crab-eating CTGGATCATTTTGGTGGCCCATACTGGTACATGAGGAAAG-

Rhesus CTGGATCATTTTGGTGGCCCATACTGGTACATGAGGAAAG-

Marmoset -----------------------------------------

**Figure 1 - figure supplement 6 - source data 2.** Multiple alignment of *PINCR* promoter region with the p53RE sequence (shown in green) was produced by the Muscle program with default parameters. The transcription start site is shown in yellow.
